# Assessment of COVID -19 associated coagulopathy and multiple hemostatic markers: a single center study in Egypt

**DOI:** 10.1007/s15010-022-01917-5

**Published:** 2022-09-22

**Authors:** Azza Abdelaal, Ahmed Abu-Elfatth, Lamees M. Bakkar, Hanan G. Abd El-Azeem, Helal F. Hetta, Eman R. Badawy

**Affiliations:** 1 Clinical Pathology Department, Faculty of Medicine, Assiut University, Assiut, 71515 Egypt; 2 Tropical Medicine and Gastroenterology Department, Faculty of Medicine, Assiut University, Assiut, 71515 Egypt; 3 Chest Department, Faculty of Medicine, Assiut University, Assiut, 71515 Egypt; 4 Department of Medical Microbiology and Immunology, Faculty of Medicine, Assiut University, Assiut, 71515 Egypt

**Keywords:** Egypt, COVID-19, Thrombosis, Coagulopathy

## Abstract

**Background:**

Coagulopathy is still a serious pattern of coronavirus-19 disease. We aimed to evaluate COVID-19-associated coagulopathy and multiple hemostatic markers in Egyptian patients. In addition, to assess coagulation acute phase reactants and its effect on the outcome.

**Methods:**

The study included 106 COVID-19 patients, and 51 controls. All patients were positive for COVID-19 infection by nasopharyngeal swab for detection of viral RNA by real-time PCR. In addition to baseline data and radiological findings, the coagulation profile was done with special attention to Fibrinogen, d-dimer, Factor VIII, von Willebrand factor (VWF), Protein C, Protein S, Antithrombin III (ATIII) and Lupus anticoagulant (LA)-1 and 2.

**Results:**

The results showed significantly higher VWF, d-dimer, and LA1 (screening) and LA2 (confirmation) in patients than a control group. Significantly higher d-dimer FVIII, VWF and LA1-2 were detected in the severe group. ATIII had high diagnostic accuracy in severity prediction. We found a significantly higher international randomized ratio (INR) and VWF among patients with thrombotic events. For prediction of thrombosis; VWF at cutoff > 257.7 has 83.3% sensitivity and 83.3% specificity.

**Conclusion:**

Patients with COVID-19 infection are vulnerable to different forms of coagulopathy. This could be associated with poor outcomes. d-Dimer is a chief tool in diagnosis, severity evaluation but not thrombosis prediction. Early screening for this complication and its proper management would improve the outcome.

## Introduction

Coagulopathy is still a serious, not well-understood pattern of coronavirus-19 disease. Its form and severity could determine the path of the patient in the hospital and his outcome [1, 2]. While d-dimer is proven to reflect the severity and the prognosis of COVID-19 patients, prothrombin time (PT), international normalized ratio (INR), partial thromboplastin time (APTT) and fibrinogen role is not well-defined [2, 3].

Immunothrombosis-associated COVID-19 infection is suggested to be relevant to the pathogenesis of COVID-associated coagulopathy (CAC) [4]. The prothrombotic state associated with COVID-19 infection is due to alterations in coagulation and immune cell malfunction. As the increased multimeric von Willebrand factor released from the injured endothelial cell (endotheliopathy) is leading to a potential increase of platelet adhesion to the endothelium [5], by the same principle, the hemostatic imbalance is augmented by the reduction of anticoagulant proteins on the surface of the injured endothelial cells. Soluble coagulation markers, including plasminogen activator inhibitor-1 and tissue factor, show marked dysregulation contributing to COVID-19-induced coagulopathy. Increased platelet-neutrophil and -monocyte aggregates induced by Platelet hyperreactivity are an adding factor for the coagulopathy observed during COVID-19. Cytokine storm associated with the COVID-19-infection promotes neutrophils to release neutrophil extracellular traps, that in turn trap platelets and prothrombotic proteins contributing to thrombotic complications [5].

As the endothelial cell injury is a constant feature in the pathogenesis of COVID-19 [2, 3], Von Willebrand factor (VWF) and factor VIII (FVIII) excess, release and degree of elevation could be a good diagnostic and prognostic marker of the disease [6–9]. Lupus anticoagulant is reported to associate with the COVID-19 infection but still its frequency, significance, the relation of its existence to other inhibitor like CRP [10–16] are debatable. Furthermore, acquired deficiency of the natural inhibitors of coagulation like Protein C (PC), Protein S (PS), antithrombin III (ATIII) could be explored as a possible contributor to the hypercoagulable state of the COVID-19 patients, their value in the prognosis and as a replacement therapy is also controversial [17–22]. This study aimed to evaluate the COVID-19-associated coagulopathy, thrombosis and disseminated intravascular coagulopathy (DIC) among patients of Upper Egypt. In addition, to assess the coagulation acute phase reactants i.e., VWF, FVIII, and acquired thrombophilia in the same patients and correlate all of them to the severity and the outcome of the patients.

### Methods

Ethics statements: This observational cross-sectional study was performed in Assiut University Hospitals; Alraghy quarantine hospital, Clinical Pathology Department during the period from May to July 2020. This study was performed in accordance with the Declaration of Helsinki and was approved by the ethics committee and institutional review board of Assiut University college of medicine (IRB no: 17300413). Informed consent was taken from all participants prior to the enrollment.

The study included 106 COVID-19 patients from critical care and general wards, and 51 age and sex-matched apparently healthy controls in the same period.

All patients were positive for COVID-19 infection by nasopharyngeal swab for detection of viral RNA by real-time PCR for SAR-COV-2 RNA assay on 7500 Applied Bio-system. Full detailed history including age, sex, comorbidities, medical history and treatment at admission were recorded. Chest computerized tomography (CT) for all patients and pulmonary CT angiography was performed when pulmonary embolism was suspected. Venous blood samples were withdrawn at admission and sent immediately to the laboratory. The following routine investigations were performed on fresh samples of patients and controls; Complete Blood Count (CBC) was performed on Pentra 80 Horiba blood counter. Liver function test, Kidney function tests, C Reactive Protein (CRP) were performed on Dimension RXL automated blood chemistry analyzer. Ferritin level on Advia 1800. Coagulation Samples were collected in a 0.109 mol/l (3.2%) sodium citrate vacutte tube, centrifuged at 3000 rpm for 15 min. PT, INR, aPTT and fibrinogen were performed on fresh samples of patients and controls by Coagulation/photo-optical method using Thromborel S, Pathromtin SL and Dade thrombin reagents respectively on Sysmex CA-1500, Siemens. d-Dimer was performed by immune-turbidity method using Innovance d-dimer reagent on Sysmex CA-1500, Siemens. Platelet poor plasma of patients and controls were stored in –80 ºC freezer, for later estimation of Factor VIII by Coagulation/photo-optical using coagulation factor VIII deficient plasma and VWF by immunoturbidity using VWF Ag reagent on Sysmex CS-2100, Siemens. PC, and ATIII were done by a chromogenic method using Berichrom Protein C, and Berichrom ATIII reagents respectively on Sysmex CA-1500, Siemens. PS was done by coagulation method on Sysmex CA-1500. Lupus anticoagulant LA1 and LA2 were done by coagulation/photo-optical method using LA1 screening reagent (DRVVT) and LA2 confirmation reagent (Platelet neutralization) respectively on System CA-1500, Siemens and LA1/LA2 ratio was calculated.

### Statistical analysis

Data were collected in a preformed data collection form before being entered into the spreadsheet. Statistical analysis was performed using the statistical package for social sciences (SPSS), version 20.0, for Windows (SPSS Inc, Chicago, IL). Continuous variables were expressed as mean and standard deviation and compared with Student’s *t* test, whereas categorical data were expressed as numbers and percentages and compared by *Chi*^*2*^ test. Correlation between C-reactive protein and LA-1, and LA-2 was assessed by Pearson correlation. Diagnostic performance of VWF (for COVID-19 diagnosis, prediction of COVID severity and prediction of thrombotic events), LA-1 (for COVID-19 diagnosis, and prediction of COVID severity) and ATIII (for diagnosis of COVID-19) were determined by receiver operator characteristics curve (ROC) by using MedCalc, version 14 (MedCalc Software, Mariakerke, Belgium). Level of confidence was kept at 95%; hence, *P* value was significant if < 0.05.

## Results

### General characteristics of enrolled patients

The mean age of enrolled patients was 45.21 ± 18.98 years. 52.8% of the patients were females. Patients were divided according to severity in a non-severe group. Severe group includes patients with clinical signs of pneumonia plus one of the following: respiratory rate > 30 breath/min, severe respiratory distress, or SpO_2_ < 90% on room air [23].

Based on the severity of the disease of the enrolled patients; 20 (18.9%) patients had a severe disease while 86 (81.1%) patients had a non-severe disease. Thrombotic events in form of pulmonary embolism occurred in 12 (11.3%) patients (Table 1).

**Table 1:** General characteristics of the enrolled COVID-19 patients

	*N* = 106
Age (years)	45.21 ± 18.98
Age group	
< 40 years	57 (53.7%)
≥ 40 years	49 (46.3%)
Sex	
Male	50 (47.2%)
Female	56 (52.8%)
Diabetes mellitus	25 (23.6%)
Hypertension	17 (16%)
Severity	
Non-severe	86 (81.1%)
Severe	20 (18.9%)
Thrombotic events	12 (11.3%)
Hospital stay	11.67 ± 7.45
Outcome	
Alive	86 (81.1%)
Died	20 (18.9%)

Data expressed as frequency (percentage), mean (SD)

### Coagulation profile among studied groups (COVID-19 patients vs. controls)

**Fig. 1:**
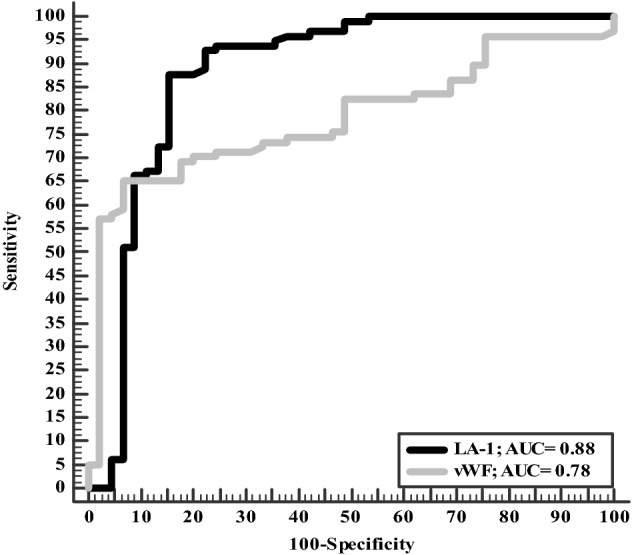
Accuracy of VWF and LA-1 in diagnosis of COVID-19

A significantly higher activated partial thromboplastin time (aPTT), VWF, d-dimer, and LA was observed among the study group (Table 2). For diagnosis of COVID-19; LA-1 has better diagnostic accuracy than VWF (87.1% vs. 73.8%) with AUC = 0.88 (Table 3, Fig. 1).

**Table 2:** Coagulation profile among studied groups (COVID-19 patients vs. controls)

	Patients group (*n* = 106)	Control group (*n* = 51)	*P* value
INR	2.05 ± 0.86	0.96 ± 0.07	0.44
aPPT (s)	35.22 ± 13.65	29.90 ± 12.26	0.01
Fibrinogen (g/l)	3.67 ± 1.48	3.35 ± 0.72	0.15
d-Dimer (mg/l)	14.56 ± 8.98	0.45 ± 0.11	< 0.001
Factor VIII (%)	150.41 ± 91	131.01 ± 70.77	0.19
Von Willebrand factor (%)	236.69 ± 69	127.19 ± 60.67	< 0.001
Protein *C* (%)	111.90 ± 42.13	108.25 ± 24.39	0.56
Protein *S* (%)	57.75 ± 30.14	60.45 ± 10.45	0.87
Antithrombin III (%)	106.83 ± 21.55	107.45 ± 19.28	0.86
Lupus anticoagulant-1 (s)	62.90 ± 20.94	29.82 ± 13.45	< 0.001
Lupus anticoagulant-2 (s)	50.20 ± 13.70	36.45 ± 8.23	< 0.001
LA-1/LA-2 ratio	1.23 ± 0.18	1.13 ± 0.34	0.03

Data expressed as mean (SD)

*s* second, *INR* international randomized ratio, *aPPT* activated partial thromboplastin time, *LA* lupus anticoagulant

*P* value was significant if  < 0.05

**Table 3:** Diagnostic accuracy of LA and VWF for diagnosis of COVID-19

	VWF	LA-1
Sensitivity	65%	88%
Specificity	93.3%	85%
PPV	96%	93%
NPV	54%	76%
Accuracy	73.8%	87.1%
Cutoff point	200	45.5
AUC	0.78	0.88

*AUC* area under the curve, *PPV* positive predictive value, *NPV* negative predictive value

### Baseline characteristics of the studied COVID-19 patients based on severity

Frequency of diabetes mellitus and hypertension was higher among the severe group. Serum ferritin level was significantly higher among the severe group (940 ± 324.97 vs. 341.86 ± 123.45; *P* < 0.001). Other data are summarized in Table 4.

**Table 4:** Characteristics and laboratory data of the COVID-19 patients based on severity

	None-severe group	Severe group	*P* value
	(*n* = 86)	(*n* = 20)	
Age (years)	40.23 ± 18.77	56.50 ± 17.01	< 0.001
Age group			< 0.001
< 40 years	54 (62.8%)	3 (15%)	
≥ 40 years	32 (37.2%)	17 (85%)	
Male sex	39 (45.3%)	11 (55%)	0.29
Diabetes mellitus	16 (18.6%)	9 (45%)	0.01
Hypertension	9 (10.5%)	7 (35%)	0.01
Cardiac disease	5 (5.8%)	3 (15%)	0.17
Hemoglobin (mg/dl)	12.99 ± 1.39	11.77 ± 2.17	< 0.001
Leucocytes (10^3^/μl)	6.25 ± 3.69	8.67 ± 4.18	0.01
Platelets (10^3^/μl)	268.26 ± 113.25	211.26 ± 115.98	0.06
Lymphocyte (10^3^/μl)	1.92 ± 0.80	2.45 ± 1.61	0.13
RDW (%)	10.66 ± 4.90	13.18 ± 3.23	0.01
C-reactive protein (mg/dl)	66.10 ± 23.45	124.94 ± 12.34	0.07
Ferritin (ng/ml)	341.86 ± 123.45	940 ± 324.97	< 0.001
Alanine transaminase (μm/l)	38.64 ± 4.95	37.54 ± 7.88	0.92
Alanine transaminase (μm/l)	31.06 ± 2.85	52.93 ± 10.51	0.06
Urea (mg/dl)	16.12 ± 2.44	8.72 ± 1.69	0.12
Creatinine (mg/dl)	60.31 ± 5.80	97.14 ± 14.20	0.25

Data expressed as frequency (percentage), mean (SD)

*RDW* red cell distribution width

*P* value was significant if  < 0.05

### Coagulation profile among enrolled COVID-19 patients

**Fig. 2:**
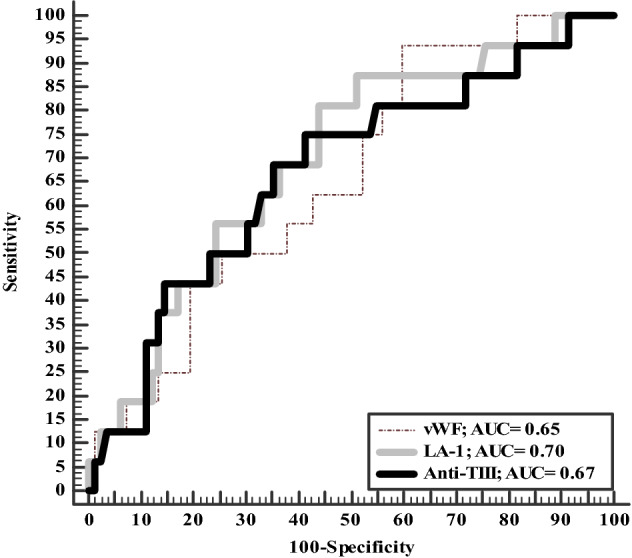
Accuracy of LA-1, ATIII, and VWF for prediction of COVID-19 severity

Severe group has significantly higher INR, aPPT, factor VIII, VWF, LA1 and LA2 and lower ATIII. Level of d-dimer was significantly higher among a severe group (16.45 ± 5.45 vs. 10.45 ± 2.34; *P* < 0.001) (Table 5). ATIII has higher diagnostic accuracy for prediction severity of COVID-19 (62.3%) in comparison to VWF (52.3%) and LA-1 (59%) 9 (Table 6, Fig. 2).

**Table 5:** Coagulation profile among studied COVID-19 patients

	None-severe group (*n* = 86)	Severe group (*n* = 20)	*P* value
INR	1.03 ± 0.15	1.34 ± 0.63	< 0.001
Prothrombin time (s)	12.98 ± 4.06	13.23 ± 2.36	0.79
Prothrombin concentration (%)	96.84 ± 31.89	90.64 ± 29.55	0.43
aPPT (s)	32.99 ± 5.75	45.03 ± 27.77	< 0.001
Fibrinogen (g/l)	3.55 ± 1.42	4.20 ± 1.68	0.08
d-Dimer (mg/l)	10.45 ± 2.34	16.45 ± 5.45	< 0.001
Factor VIII (%)	134.53 ± 79.65	216.33 ± 110.51	< 0.001
von Willebrand factor (%)	219.26 ± 104.87	327.13 ± 60.04	< 0.001
Protein C (%)	113.47 ± 35.78	105.14 ± 63.43	0.42
Protein S (%)	59.49 ± 31.19	49.13 ± 23.47	0.20
Antithrombin III (%)	109.53 ± 18.39	95.24 ± 29.63	< 0.001
Lupus anticoagulant-1 (s)	58.10 ± 13.37	85.33 ± 33.45	< 0.001
Lupus anticoagulant-2 (s)	46.98 ± 7.01	65.10 ± 24.11	< 0.001
LA-1/LA-2 ratio	1.22 ± 0.17	1.86 ± 0.25	< 0.001

Data expressed as mean (SD)

*s* second,* INR* international randomized ratio, *aPPT* activated partial thromboplastin time, *LA* lupus anticoagulant

*P* value was significant if  < 0.05

**Table 6:** Accuracy of LA-1, VWF and ATIII in prediction of COVID-19 severity

	VWF	LA-1	ATIII
Sensitivity	75%	75%	55%
Specificity	47%	55.3%	64%
PPV	21%	28%	26%
NPV	91%	90.4%	86%
Accuracy	52.3%	59%	62.3%
Cutoff point	203	58.4	104
AUC	0.65	0.70	0.67

*AUC* area under the curve, *PPV* positive predictive value, *NPV* negative predictive value

#### Outcome and hospital stay among studied COVID-19 patients based on severity

Six (30%) patients of the severe group and 5 (5.8%) patients of the non-severe group developed thrombotic events in form of pulmonary embolism. Hospital stay was more prolonged among the severe group. All patients were improved with exception of one patient in a non-severe group and 14 (70%) patients in the severe group (Table 7).

**Table 7:** Outcome and hospital stay among studied COVID-19 patients

	None-severe group (*n* = 86)	Severe group (*n* = 20)	*P* value
Thrombotic events	8 (9.3%)	4(20%)	< 0.16
Hospital stay (days)	10.25 ± 8.34	14.84 ± 7.1745	0.01
Outcome			< 0.001
Alive	85 (98.85%)	6 (30%)	
Died	1 (1.2%)	14 (70%)	

Data expressed as frequency (percentage), mean (SD)

*P* value was significant if < 0.05

### Coagulation profile based on thrombotic events

There were significantly higher INR (2.16 ± 0.81 vs. 1.05 ± 0.19; *P* < 0.001) and VWF (310.37 ± 68.37 vs. 228.41 ± 106.51; *P* = 0.02) among those with thrombotic events (Table 8).

**Table 8:** Coagulation profile based on thrombotic events

	Thrombotic events (*n* = 12)	No-thrombotic events (*n* = 94)	*P* value
INR	2.16 ± 0.81	1.05 ± 0.19	< 0.001
aPPT (s)	35.76 ± 16.91	35.16 ± 13.32	0.89
Fibrinogen (g/l)	3.94 ± 1.44	3.64 ± 1.49	0.52
d-Dimer (mg/l)	15.75 ± 6.67	13.67 ± 7.56	0.90
Factor VIII (%)	197.35 ± 107	144.79 ± 88.82	0.07
Von Willebrand factor (%)	310.37 ± 68.37	228.41 ± 106.51	0.02
Protein C (%)	115.29 ± 68.80	111.51 ± 38.44	0.78
Protein S (%)	63.79 ± 23.41	57.18 ± 30.87	0.58
Antithrombin III (%)	100.03 ± 20.66	107.62 ± 21.61	0.27
Lupus anticoagulant-1 (s)	69.81 ± 15.79	62.08 ± 21.39	0.25
Lupus anticoagulant-2 (s)	55.46 ± 12.01	49.63 ± 13.81	0.20
LA-1/LA-2 ratio	1.26 ± 0.08	1.23 ± 0.19	0.63

Data expressed as mean (SD)

*s* second, *INR* international randomized ratio, *aPPT* activated partial thromboplastin time, *LA* lupus anticoagulant, *aPPT* activated partial thromboplastin

*P* value was significant if < 0.05

**Fig. 3:**
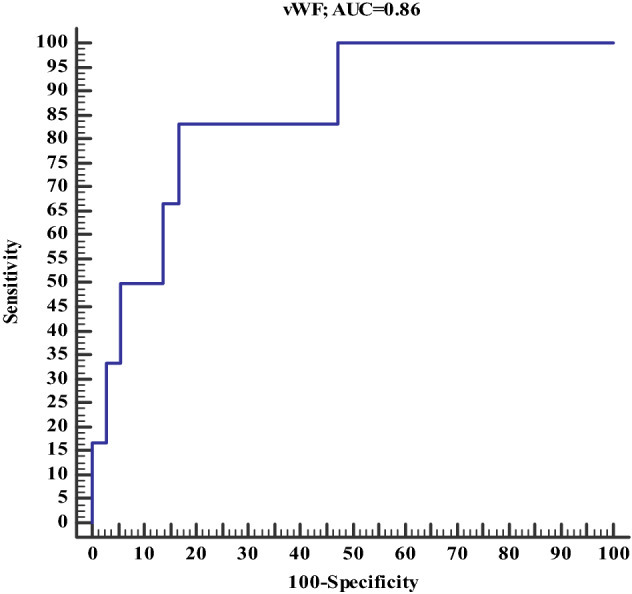
Accuracy of VWF in prediction of thrombotic events in COVID-19

For prediction of thrombotic events in such patients; VWF at cutoff > 257.7 has 83.3% sensitivity, 83.3% specificity with overall accuracy was 83.3% and area under the curve (AUC) was 0.86 (Table 9, Fig. 3).

**Table 9:** Accuracy of VWF in prediction of thrombotic events

	VWF
Sensitivity	83.3%
Specificity	83.3%
Positive predictive value	45.5%
Negative predictive value	97%
Accuracy	83.3%
Cutoff point	257.7
Area under the curve	0.86

**Fig. 4:**
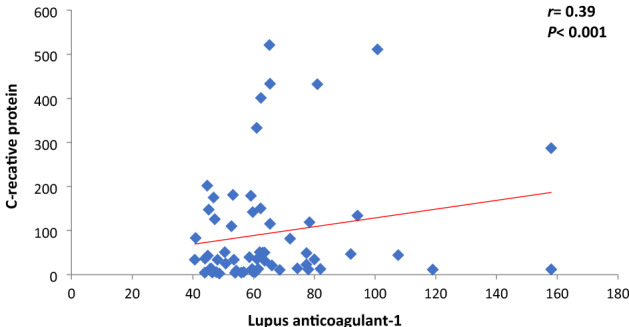
Correlation between LA-1 and CRP among COVID-19 patients

**Fig. 5:**
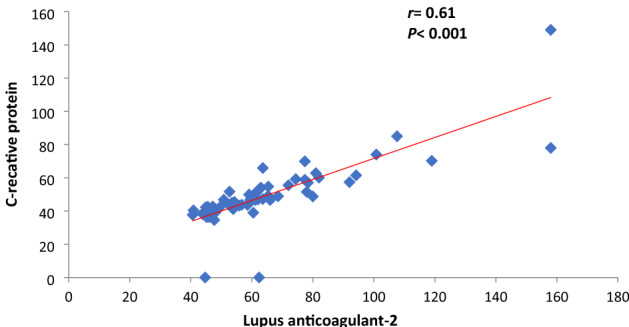
Correlation between LA-2 and CRP among COVID-19 patients

Correlation between CRP and LA among COVID-19 patients: both LA-1 and LA-2 had significant positive correlation with CRP (*r* = 0.39, *P* < 0.001 with LA-1 and *r* = 0.61, *P* < 0.001 for LA-2) (Figs. 4, 5)

## Discussion

We aimed in this study to evaluate the COVID-19-associated coagulopathy and DIC among patients of Upper Egypt. The study included 106 COVID-19 patients and 51 apparently healthy controls. Twenty (18.9%) of the patients had a severe disease while 86 (81.1%) patients had a non-severe disease. We found that patients with severe disease had significantly higher age with a higher frequency of diabetes mellitus and hypertension. Serum ferritin and CBC; leucocytes, RDW were significantly higher among a severe group.

### Coagulation screen and d-dimer

aPTT, d-dimer results were significantly higher among the patients. Fibrinogen was more elevated in a severe group but not yet statistically significant. Severe group has significantly prolonged INR, aPPT and d-dimer.

According to the American society of hematology (ASH) COVID-19 resources, the COVID -19 associated coagulopathy is commonly presented with a slight increase in PT/aPTT with more elevations in fibrinogen and d-dimer. In addition, there is a corresponding increase in inflammation markers e.g., CRP [2]. Gomez-Mesa et al. described COVID-19-associated coagulopathy as a mixture of a mild form of DIC and pulmonary thrombotic microangiopathy that might intensely determine the prognosis of the disease [1].

Some reports showed significantly higher d-dimer and FDP levels, longer PT and aPTT in COVID-19 non-survivors compared to survivors at patient’s admission [24, 25]. In other reports, 20–50% of hospitalized patients was described to have increased d-dimer, prolonged PT, thrombocytopenia, and/or low fibrinogen levels with more thrombotic than hemorrhagic events [1].

DIC is typically characterized by the concurrent occurrence of widespread thrombosis, contributing to organ failure. Depletion of coagulation factors and platelets may occur, resulting in overt bleeding from various sites. DIC is constantly happens on top of an underlying disorder, such as malignancies, trauma, or obstetric complications [26].

Seventy-eight (73.6%) of our total cases had changes in their basic coagulation profile that range from mild prolongation of PT and or aPTT, abnormal fibrinogen level and elevated d-dimer up to severe changes in form of highly elevated d-dimer or full criteria meeting overt DIC-ISTH lab score [27]. Among the 78 patients, 16 had low fibrinogen level and 39 patients had high fibrinogen level.

Ferrri et al. Stoichitoiu et al. are other studies did not find a significant change in fibrinogen levels according to severity or association with harmful outcome [18, 21]. Yet, fibrinogen is still a valuable marker because high fibrinogen level can differentiate between COVID-19-associated coagulopathy from the hypofibrinogenemia [27] associated with DIC in COVID-19 patients.

Concerning platelet count, COVID-19 associated coagulopathy is depicted by marked elevation of d-dimer deprived of thrombocytopenia or a clotting times impairment, which suggests a process of thrombin generation and local fibrinolysis [1]. Our results as in previous studies suggest that COVID-19-associated coagulopathy/DIC thrombocytopenia is low compared to sepsis-induced coagulopathy/DIC [2]. Additionally, we did not find a significant difference in platelets count associated with increased risk of disease severity and mortality.

d-Dimer elevation is a chief marker that is correlated to COVID-19-associated coagulopathy diagnosis, severity, risk of intensive care unit (ICU) and mortality [7]. Many studies approved the significance of d-dimer, for instance, Han et al. have studied 94 COVID-19 patients. d-Dimer was (10.36 vs. 0.26 ng/l; *P* < 0.001) in patients compared to healthy controls respectively and the rise in d-dimer value was more significant in critically ill patients [25].

Guan et al. studied a large COVID-19 sample (1099 patients) in whom he found d-dimer levels elevated in 43% of the non-severe patients vs. 60% in intensive care unit (ICU) patients [28]**.** Other studies linked the increase in d-dimers at admission, without signs DIC, to increased risk of death [29, 30]. In our study, 57% of all patients had elevated d-dimer at admission, 45% in the severe group, and the percentage among non survivors was 80%.

Tang et al. reported that only 0.6% of the COVID-19 survivors in his study met the criteria of DIC while 71.4% of non-surviving patients have fulfilled the criteria [24]. Amid the non-survivors, the score in the DIC parameters was as follows; 85.7%, 47.6%, 28.6%, 23.8% for d-dimer, PT, fibrinogen and platelet count respectively and explained it as secondary hyperfibrinolysis following the coagulation activation. In contrary to this data, in our study only two patients (13.3%) of the non survivors and one patient (1.09%) among survivors have developed DIC. Both DIC non-survivor patients had 6 ISTH lab score and Liver cirrhosis and were among the severe group and one of them suffered also from sepsis [24]. The survivor patient who developed DIC was belonged to a non-severe group and had a score of 5 by ISTH criteria. Noticeably none of the three patients had overt bleeding symptoms.

### Acute-phase reactants (FVIII and VWF)

In our study, VWF results are significantly higher among the patients compared to controls. Both VIII and VWF are significantly higher among the severe group. Our result is consistent with Rauch A et al. who proved that coagulation biomarkers are associated with the severity of COVID-19. In addition to VWF, they recognized that CRP, d-dimers, fibrinogen, levels were highest for patients straightforwardly admitted to the ICU rather than FVIII [8].

Likewise, Philippe et al. studied 4 different endothelial markers for the prediction of COVID-19 hospital mortality and identified VWF:Ag as the best of them. In addition, VWF Antigen (VWF:Ag) in his patients was significantly higher compared to non-COVID-19 patients and in critical patients to non-critical patients [7]. In our study, we had 4 patients whose VWF:Ag exceeded 600%, which is the upper border of the instrument linearity. All of them were among the severe group, two of them died and one of the non-survivors had PE.

### Acquired thrombophilia (LA, PC, S, ATIII)

Most of the studies confirmed a high prevalence of acquired thrombophilia in COVID-19 patients [10, 18–22, 31]. The data are contradictory about its significance and relation to the disease severity and prognosis.

First, as regard to LA; Gazzaruso et al. found that LA is common among COVID-19 patients and it may be an adverse effect of severe inflammation rather than a cause of thrombosis [10]. Vollmer et al. stated that LA was frequent in COVID-19 in the acute phase but with controversial significance and that it was transient and strongly associated with thrombosis [31]. Pineton et al. found that patients were positive for LA which is the most anti phospholipid antibody (aPLA) strongly associated with thrombosis [14]. Alternatively, Galeano-Valle et al. showed that antiphospholipid antibodies (aPLA) are not frequent in COVID-19 patients [32].

In our study, only five (4.7%) patients are considered LA positive by DRVVT (LA1 is prolonged, LA2 is normal with LA1/LA2 ratio > 1.2) vs. 4 subjects out of 51 healthy control group. Most of patients, 83 patients (78%) had prolonged LA1, LA2 and ratio. Interestingly the degree of prolongation of LA1 is striking as most of the samples were between 60–100 s.

In a previous study, LA was found in up to 90% of patients associated with prolonged aPTT [12]. We did not find a similar correlation between LA with prolonged aPTT. We found both LA-1 and LA-2 had a significant positive correlation with CRP (*r* = 0.39, *P* < 0.001 with LA-1 and *r* = 0.61, *P* < 0.001 for LA-2) similar to the previous study [11] and we agree to Pineton de Chambrun et al. [14] that CRP may be a cause of false positive LA.

Second, with regard to other causes of acquired thrombophilia, Ferrari et al. [18] detected a 20% prevalence of PS deficiency, again, without prognostic significance. Stoichitoiu et al. found that 59(65%) out of 91 patients had low PS^.^ level and was negatively correlated with clinical severity and lung damage in CT. PC wasn’t associated with harmful outcome [21].

ATIII deficiency was observed in COVID-19 patients as described in case series and case reports [19–22] with curious concerns about the efficacy of Heparin with patients of ATIII deficiency. Gazzaruso and his colleagues also correlated ATIII with poor outcomes and found its level in non-survivor significantly lower than survivors [19]. In our study, no significant difference was found between COVID-19 patients and normal controls with regard to Protein C, S and ATIII levels, with a 60% cut off for PS similar to Stoichitoiu et al. study [21]. Only three patients of ours had ATIII < 70% (3 of the DIC patients) but we found a significant decrease in ATIII levels in a severe group than non-severe COVID-19 patients(95.24 ± 29.63 vs. 109.53 ± 18.39; *P* < 0.001).

### Thrombotic marker

The high incidence of VTE in COVID-19 particularly critically ill patients is supported by several studies [2]. The incidence of thrombosis in COVID-19 ICU patients is described to be 20% to 30% [33, 34]. Tan et al. evaluated 102 studies and estimated 14.7% for VTE which was significantly higher in intensive care unit (ICU) and 0.9–3.9% for ATE [35].

Large percentage of the patients who received a therapeutic dose of anticoagulation developed thromboses is considered evidence that the abnormal hemostasis seen in COVID-19 patients are fairly distinctive [21]. Many assumptions were postulated to explain the pathology and risk factors of VTE in COVID-19 patients. LA was found to be independently associated with thrombosis [36], Hypoxia and IL6 lead to a severe decrease in PS that might aggravate the thrombosis risk [17]. Acute phase reactants; FVIII, VWF, fibrinogen and higher d-dimer value are examples [1].

We had 8 patients (9.3%) in the non-severe group vs. 4 patients (20%) among the severe group who developed VTE. All in form of PE, ranging from sub-segmental to main branch PE by CT angiography. Comparing the coagulation profile results of the group of patients who developed PE with the rest of the patients, we found significantly higher INR among those with thrombotic events.

The cause of higher INR among the thrombosis group is not clear as only one patient was on Marian therapy but could be the presence of other combined and long-term comorbidities like DM, hypertension and cardiac diseases. In addition, 2 of the thrombotic patients have a history of hepatitis C virus infection. Our results are highly consistent with the idea that acute respiratory distress syndrome and thrombotic complications of COVID-19 could be elucidated through VWF-related mechanism [6]. On contrary to Rauch A et al. results who did not find VWF levels at admission predictive of thrombotic events [8].

Finally, we used the ROC curve to estimate a cut-off value for the diagnosis of COVID-19; LA-1 has better diagnostic accuracy than VWF (87.1% vs. 73.8%) with AUC was 0.88. For prediction severity of COVID-19, ATIII has higher diagnostic accuracy (62.3%) in comparison to VWF (52.3%) and LA-1 (59%). For prediction of thrombotic events in such patients; VWF at cutoff > 257.7 has 83.3% sensitivity, 83.3% specificity with overall accuracy was 83.3% and area under the curve (AUC) was 0.86.

### Limitation of this study

Could be the lack of follow-up samples during the progress of the disease to identify any persistence of impaired parameters and corelate them with deleterious effects throughout the course of the disease. Although prolonged results of the most patient for LA1 did not require enhancement of sensitivity by another screening test with different principal, absence of the sample mixing with normal plasma did not allow us to further recognise the causes behind the prolongation of LA1 test. Furthermore, lack of correlation between the comorbidities and abnormal hemostatic profile. Finally, not all coagulation parameters are readily available in all hospitals.

## In conclusion

Our results are confirming the relationship between COVID-19-associated coagulopathy with severity and poor outcome of the patients; prolonged INR, aPPT and d-dimer are the bad prognostic markers. d-Dimer elevation at admission is a chief tool in diagnosis, and severity evaluation but not for prediction of thrombosis. Patients with COVID-19 should be tested during their hospital stay, especially if were admitted to the intensive care unit, for coagulation disorders. Future comparative studies to assess the severity and range of coagulopathy in patients with COVID-19 infection and patients without COVID-19 infection.

## Data Availability

All generated are included in this manuscript.
